# Pri-miR-124 rs531564 and pri-miR-34b/c rs4938723 Polymorphisms Are Associated with Decreased Risk of Esophageal Squamous Cell Carcinoma in Chinese Populations

**DOI:** 10.1371/journal.pone.0100055

**Published:** 2014-06-19

**Authors:** Junjie Zhang, Xuewen Huang, Juanjuan Xiao, Yajun Yang, Yinghui Zhou, Xiaofeng Wang, Qingmei Liu, Jingmin Yang, Mengyun Wang, Lixin Qiu, Yabiao Zheng, Ping Zhang, Jin Li, Ya’nong Wang, Qingyi Wei, Li Jin, Jiucun Wang, Minghua Wang

**Affiliations:** 1 Department of Biochemistry and Molecular Biology, Medical College, Soochow University, Suzhou, Jiangsu, China; 2 Clinical laboratory, Huadong Sanatorium, Dajishan, Meiyuan Garden, Wuxi, Jiangsu, China; 3 MOE Key Laboratory of Contemporary Anthropology and State Key Laboratory of Genetic Engineering, School of Life Sciences, Fudan University, Shanghai, China; 4 Cancer Research Laboratory, Fudan University Shanghai Cancer Center, Shanghai, China; 5 Department of Oncology, Shanghai Medical College, Fudan University, Shanghai, China; 6 Department of Medical Oncology, Fudan University Shanghai Cancer Center, Shanghai, China; 7 Department of Gastric Cancer & Soft Tissue Sarcoma Surgery, Fudan University Shanghai Cancer Center, Shanghai, China; University of Central Florida, United States of America

## Abstract

MicroRNAs are a new class of small non-protein-coding RNAs that sometimes function as tumor suppressors or oncogenes. Aberrant expression and structural alteration of microRNAs have been reported to be involved in tumorigenesis and cancer development. Recently, rs531564/pri-miR-124-1, rs4938723/pri-miR-34b/c, rs7372209/pri-miR-26a-1, rs895819/pre-miR-27a, and rs11134527/pri-miR-218 were reported to be associated with risks of various cancers. In order to evaluate the relationship of these SNPs and esophageal squamous cell carcinoma (ESCC) risk, we conducted a case-control study with 1109 ESCC patients and 1275 control subjects to examine the potential association of these pri/pre-miRNA polymorphisms with ESCC susceptibility. As a result, two SNPs were associated with a significant risk of ESCC. We found that the GG genotype of pri-miR-124-1 rs531564 was associated to a significantly decreased risk of ESCC comparing with the CC/CG genotypes (p = 0.005; OR = 0.61, 95% CI = 0.43–0.86). In addition, the CC genotype of pri-miR-34b/c rs4938723 was associated with a significant decreased risk of ESCC (CC VS. TT/TC: p = 0.007, OR = 0.82, 95% CI = 0.71–0.95) in Chinese population. The present study provides the first evidence that pri-miR-124-1 rs531564 and pri-miR-34 rs4938723 were associated with the risk of ESCC in Chinese population.

## Introduction

Esophageal cancer is considered one of the most aggressive cancers in the world. It includes two main types: esophageal adenocarcinoma (EADC) and esophageal squamous cell carcinoma (ESCC) [1]. ESCC is the predominant type of esophageal cancer in the East Asian population [2]. Epidemiological studies indicate that unhealthy lifestyles, including smoking tobacco and drinking alcohol, are major risk factors for esophageal cancer. However, only a subset of individuals would develop esophageal cancer under the environmental risk factors. This suggests the role of risk factors and genetic alterations in esophageal cancer carcinogenesis through gene-environment interactions. A series of single nucleotide polymorphisms (SNPs) were found to be associated with the risk of ESCC in studies with different designs and populations, and results were not consistency. It indicated that the genetic etiology of ESCC is complicated, and further independent investigation is needed to confirm the associations with ESCC risk.

MicroRNAs (miRNAs) are small endogenous 19–25 nucleotide (nt) non-coding RNAs that regulate gene expression by base pairing with target mRNAs at the 3-untranslated region, leading to mRNA cleavage or translational repression [3], [4]. By bioinformatic method, a single miRNA is predicted to bind to a large number of mRNA targets [5]. MiRNAs have been suggested to regulate almost one-third of human genes and most cancer-linked genes [6]. The biogenesis of miRNA proceeds as follows: a miRNA gene is transcribed into a primary miRNA (pri-miRNA) covering several hundred or thousand nucleotides in length [7], [8]. The pri-miRNA then becomes a precursor miRNA (pre-miRNA) of 60–70 nt in length [9], [10]. Finally, the pre-miRNA is processed to a mature miRNA in length of 21–25 nt [11], [12].

SNPs or mutations in miRNA genes can influence the processing and/or target binding of miRNAs, thus resulting in diverse functional consequences and thereby possibly representing potential candidate biomarkers for cancer prognosis [13]–[15]. MiR-124, including miR-124-1, functions as suppressors of hepatocellular carcinoma, hematological malignancies, cervical cancer and gastric cancer [16]–[19]. Recently, rs531564 with a G/C variation was identified in pri-miR-124-1. This G/C variant has been reported to affect the expression of mature miR-124 in the central nervous system [20]. This SNP was found associated with risks of gastric cancer, bladder cancer, and esophageal adenocarcinoma [21]–[23]. Apart from this microRNA, there are many other microRNAs that have been identified as functional factors in cancer generation. For example, on the one hand, rs4938723/pri-miR-34b/c is associated with a significantly increased risk of hepatocellular carcinoma [24], while on the other hand, it significantly decreased the risk of colorectal cancer [25]. Rs895819/pre-miR-27a is associated with a significantly reduced risk of renal cell cancer [26]. Rs7372209/pri-miR-26a-1 decreased bladder cancer risk in women [23], and rs11134527/pri-miR-218 is associated with a significantly decreased risk of cervical carcinoma [27], [28]. On the basis of the biological and pathologic significance of miRNA, it is possible that functional genetic variations in the pre/pri-miRNAs may contribute to the development of ESCC.

There were some studies related to these SNPs and cancer risks. However, whether these genetic variants of miRNA-related genes have an influence on the risk of ESCC has largely remained unknown. We have conducted a case-control study to investigate the potential association between rs531564/pri-miR-124-1, rs4938723/pri-miR-34b/c, rs7372209/pri-miR-26a-1, rs895819/pre-miR-27a, rs11134527/pri-miR-218 and the risk of ESCC.

## Methods

### Ethics Statement

The study was approved by the Institutional Review Board of the School of Life Sciences of Fudan University. Moreover, all participants had signed a written informed consent for donating their biological samples.

### Study Population

These cases (age range = 37–88 years) were newly diagnosed patients, with histologically confirmed ESCC. All cases were recruited from two hospitals in eastern China: Taizhou People’s Hospital and Shanghai Cancer Hospital between June 2009 and September 2011. There were no age, sex, ethnicity, or cancer stage restrictions on recruitment. Informed consent was provided to the patients who had been diagnosed in these hospitals during this period. Patients who agreed to the investigation were asked to fill the questionnaire.

All controls were recruited from the Taizhou Longitudinal study conducted in Taizhou City, eastern China during the same period as cases [29]. The control subjects had no history of cancer and were divided in groups according to their age and sex (we take five years as a gap for division of groups). The controls are matched in frequency with the case group.

All participants were interviewed with a structured questionnaire in order to obtain demographic information, including age and sex. Data on alcohol consumption and smoking status were also collected. After the interview, 5 mL venous blood sample was collected from each participant.

For quality control of the samples, we checked the questionnaire, the clinical information of cases and the blood sample carefully. The samples that did not match the criteria were excluded (i.e. wrongly classified esophageal cancer type, blood sample missing, etc.).

Finally, this case-control study was composed of 1109 ESCC patients of Han Chinese and 1275 unrelated cancer-free control subjects with matched age, sex and ethnics.

### SNP Selection and Genotyping

Candidate SNPs were selected based on a literature review of pri/pre-miRNA epidemiological studies [20], [21], [30]–[33]. A literature search was taken in the PubMed database. All papers reporting the association between the SNPs of miRNA genes and cancer risks were retrieved. Considering that ESCC is a type of esophageal carcinoma, as well as of squamous cell carcinoma, we have examined all the papers related to other types of esophageal carcinoma (i.e. EADC) or squamous cell carcinoma (i.e. cervical cancer) apart from ESCC. The SNPs that were already identified with clear relationship of ESCC risks were excluded. Finally, we selected these 5 candidate SNPs in our study, which are rs531564 in pri-miR-124-1, rs4938723 in pri-miR-34b/c, rs7372209 in pri-miR-26a-1, rs895819 in pre-miR-27a, and rs11134527 in pri-miR-218.

Genomic DNA was extracted from peripheral blood samples with a salt precipitation method. Genotyping was detected with a SNaPshot Multiplex System for single nucleotide extension. Loci of the above SNPs were first amplified by PCR. The PCR products were treated with SAP and ExoI to remove primers and unincorporated dNTPs, followed by ddNTP extension (SNaPshot) and fragment analysis. All primers are shown in Table S1. Results were analyzed by the Peak scanner version 1.0 (Applied Biosystems).

For the quality control of the genotyping, 5% of samples were selected randomly as replicates, and the reproducibility was 100%.

### Statistical Analysis

Differences in selected variables and Hardy-Weinberg equilibrium were evaluated using the Chi-square test and Student’s t test as appropriate. Differences in the distributions of demographic characteristics and frequencies of genotypes between patients and control subjects were evaluated using Student’s t test (for continuous variables) or Pearson’s Chi-square test (for categorical variables).

The following genetic models were used in the analysis procedure: the dominant model (AA vs AB+BB), the recessive model (AA+AB vs BB), the codominant model (AA vs AB & AA vs BB) and the overdominant model (AA+BB vs AB); all models assuming B is the risk allele.

The odds ratios (ORs) and 95% confidence intervals (CIs) were calculated by univariate and multivariate logistic regression analyses to determine associations between rs531564/pri-miR-124-1, rs4938723/pri-miR-34b/c, rs7372209/pri-miR-26a-1, rs895819/pre-miR-27a, rs11134527/pri-miR-218 genotypes and the risk of ESCC. Adjusted ORs were computed using unconditional logistic regression with adjustment for age, sex, smoking, and drinking. A p-value less than 0.05 was considered statistically significant. The statistical analyses above were performed with the SPSS Software version 12.0 (SPSS, Chicago, IL, USA) and were based on two-tailed probability.

To investigate the combined contributions of genetic and non-genetic (such as smoking and drinking) factors involved in ESCC risk, we also performed the multifactor dimensionality reduction (MDR) analysis. This method was performed by MDR V2.0 software. (http://www.multifactordimensionalityreduction.org).

## Results

### Characteristics of the Study Population

Among all the ESCC patients invited in these hospitals, approximately 90% of them are involved in this investigation. As shown in Table 1, the final analysis included 1109 ESCC patients and 1275 control subjects. No significant difference in age (p = 0.564) and sex (p = 0.229) was detected between case and control subjects, which shows that the frequency matching was adequate. However, higher rates of smokers and drinkers were identified in case subjects than in controls (p<0.001).

**Table 1:** Characteristics of case and control subjects.

Characters	Case (n = 1109)		Control (n = 1275)		p^a^
	n	%	n	%	
Age (years)					0.564
_<_62	520	46.9	582	45.6	
_≥_62	589	53.1	693	54.4	
Age (mean±SD)	62.77±8.98		63.50±8.96		0.076^b^
Sex					0.229
Male	857	77.3	958	75.1	
Female	252	22.7	317	24.9	
Smoking					<**0.001**
Yes	721	65.0	687	53.9	
No	388	35.0	588	46.1	
Drinking					<**0.001**
Yes	570	51.4	435	34.1	
No	539	48.6	840	65.9	

a Two-sided ^2^ test.

b Mann-Whitney U test.

Table 2 showed the allelic and genotype distributions of individual SNPs in the case and the control subjects. The observed frequencies for those polymorphisms were in agreement with the frequencies expected under Hardy–Weinberg equilibrium (p>0.05 for each). The genotype frequencies of pri-miR-124-1 rs531564 C>G were 72.4% (CC), 26.6% (CG), and 1.0% (GG) in the cases and 71.4% (CC), 26.0% (CG), and 2.6% (GG) in the controls, respectively; and the difference was statistically significant (p = 0.011). Table 3 showed the main effect of these SNPs and ESCC risks based on different genetic models. In the recessive model, we found that the GG genotype of rs531564 was revealed to decrease the risk of ESCC (GG VS. CC/CG, p = 0.005, OR = 0.61, 95% CI = 0.43–0.86). As the number of GG genotype is a bit small, we also take a Fisher’s Exact Test and the result is still significant (p = 0.002). In the dominant model, no significant relationship between this SNP and ESCC risk was found (GG/CG VS. CC, p = 0.477, OR = 0.94, 95% CI = 0.78–1.12).

**Table 2:** Genotyping results of the candidate SNPs.

	location	Genotype	Cases (n = 1109 )		Controls (n = 1275 )		OR (95% CI)^a^	p	p_HWE_ of control
			n	%	n	%			
miR-124-1 rs531564	primary-miRNA	CC	803	72.4	910	71.4	1	**0.011** b	0.840
		CG	295	26.6	331	26.0	0.99 (0.83, 1.20)	0.952	
		GG	11	1.0	34	2.6	0.61 (0.43, 0.87)	**0.005**	
miR-34 rs4938723	primary-miRNA	TT	489	44.1	569	44.6	1	**0.033** b	0.816
		TC	536	48.3	573	44.9	1.10 (0.93, 1.31)	0.276	
		CC	84	7.6	133	10.5	0.84 (0.72, 0.98)	**0.023**	
miR-26-1 rs7372209	primary-miRNA	GG	541	48.8	628	49.3	1	0.341^b^	0.919
		GA	454	40.9	538	42.2	0.98 (0.83, 1.17)	0.888	
		AA	114	10.3	109	8.5	1.10 (0.95, 1.28)	0.186	
miR-27 rs895819	precursor-miRNA	AA	613	55.3	719	56.4	1	0.851^b^	0.481
		AG	414	37.3	466	36.5	1.03 (0.87, 1.23)	0.724	
		GG	82	7.4	90	7.1	1.04 (0.89, 1.23)	0.625	
miR-218 rs11134527	primary-miRNA	TT	396	35.7	454	35.6	1	0.512^b^	0.517
		TC	529	47.7	630	49.4	0.94 (0.78, 1.12)	0.491	
		CC	184	16.6	191	15.0	1.06 (0.93, 1.20)	0.382	

a Adjusted for age, sex, drinking and smoking.

b Chi-square test for 3 genotypes between case and control groups.

**Table 3:** Association analyses of the candidate SNPs with ESCC risk.

	MAF		Dominant		Recessive		Overdominant	
	Case	Control	OR (95% CI)^a^	p	OR (95% CI)^a^	p	OR (95% CI)^a^	p
miR-124-1 rs531564 C>G	0.143	0.156	0.94 (0.78, 1.12)	0.477	0.61 (0.43, 0.86)	**0.005**	1.02 (0.84, 1.22)	0.867
miR-34 rs4938723 T>C	0.317	0.329	1.02 (0.87, 1.21)	0.780	0.82 (0.71, 0.95)	**0.007**	1.17 (0.99, 1.38)	0.066
miR-26-1 rs7372209 G>A	0.307	0.296	1.03 (0.87, 1.21)	0.743	1.11 (0.96, 1.27)	0.158	0.96 (0.81, 1.13)	0.615
miR-27 rs895819 A>G	0.261	0.253	1.04 (0.88, 1.23)	0.642	1.03 (0.88, 1.21)	0.707	1.02 (0.86, 1.21)	0.782
miR-218 rs11134527 T>C	0.404	0.397	0.98 (0.83, 1.16)	0.829	1.08 (0.97, 1.21)	0.170	0.90 (0.77, 1.07)	0.228

a Adjusted for age, sex, drinking and smoking.

The genotype frequencies of pri-miR-34b/c rs4938723 T>C for TT, TC and CC were 44.1%, 48.3% and 7.6% in the cases and 44.6%, 44.9% and 10.5% in the controls respectively. There was a significant difference (p = 0.033) between the cases and the controls. In the recessive model, the CC genotype was found associated with the decreased risk for ESCC (CC VS. TT/TC: p = 0.007, OR = 0.82, 95% CI = 0.71–0.95). Fisher’s Exact Test also indicates the significant result (p = 0.018). In dominant model, however, no significant result was found (CC/TC VS. TT: p = 0.780, OR = 1.02, 95% CI = 0.87–1.21).

However, other polymorphisms, pri-miR-26-1 rs7372209 G>A, pre-miR-27a rs895819 A>G and pri-miR-218 rs11134527 T>C, did not demonstrate any associations with the risk of ESCC in the codominant, dominant, recessive or overdominant models.

### Stratification Analyses of Pri-miR-124-1 rs531564 C>G, Pri-miR-34b/c rs4938723 T>C and the Risk of ESCC

To further evaluate the associations between pri-miR-124-1 rs531564 C>G, pri-miR-34b/c rs4938723 T>C and ESCC risk, respectively, we performed the stratification analyses in the recessive model according to age, sex, smoking and drinking (Table 4 and Table 5).

**Table 4:** Stratified results for pri-miR-124-1 rs531564 C>G by age, sex, drinking and smoking.

Variable	GG vs. CC+CG		Crude OR (95% CI)	p	Adjust OR (95% CI)^a^	p
	Case	Control				
Age						
<62	6/514	12/570	0.55 (0.21, 1.49)	0.341	0.76 (0.46, 1.25)	0.278
≥62	5/584	22/671	0.26 (0.10, 0.69)	0.005	0.52 (0.32, 0.86)	**0.010**
Sex						
Males	10/847	23/935	0.48 (0.23, 1.01)	0.054	0.70 (0.48, 1.02)	0.062
Females	1/251	11/306	0.11 (0.01, 0.86)	0.015	0.36 (0.13, 1.01)	0.051
Drinking						
Yes	8/562	7/428	0.87 (0.31, 2.42)	0.799	0.91 (0.55, 1.52)	0.723
No	3/536	27/813	0.17 (0.05, 0.56)	0.001	0.41 (0.23, 0.75)	**0.004**
Smoking						
Yes	9/712	18/669	0.47 (0.21, 1.05)	0.079	0.69 (0.46, 1.05)	0.081
No	2/386	16/572	0.19 (0.04, 0.81)	0.013	0.43 (0.21, 0.91)	**0.026**

a Adjusted for age, sex, drinking and smoking.

**Table 5:** Stratified results for pri-miR-34b/c rs4938723 T>C by age, sex, drinking and smoking.

Variable	CC vs. TT+TC		Crude OR (95% CI)	p	Adjust OR (95% CI)^a^	p
	Case	Control				
Age						
<62	38/482	56/526	0.74 (0.48, 1.14)	0.195	0.86 (0.69, 1.07)	0.185
≥62	46/543	77/616	0.68 (0.46, 0.99)	0.046	0.79 (0.65, 0.96)	**0.016**
Sex						
Males	66/791	99/859	0.72 (0.52, 1.00)	0.060	0.84 (0.71, 0.99)	**0.033**
Females	18/234	34/283	0.64 (0.35, 1.16)	0.146	0.75 (0.54, 1.03)	0.075
Drinking						
Yes	47/523	51/384	0.68 (0.45, 1.03)	0.069	0.81 (0.65, 1.01)	0.056
No	37/502	82/758	0.68 (0.46, 1.02)	0.063	0.83 (0.67, 1.01)	0.062
Smoking						
Yes	58/663	78/609	0.68 (0.48, 0.98)	0.038	0.81 (0.68, 0.98)	**0.026**
No	26/362	55/533	0.70 (0.43, 1.13)	0.156	0.83 (0.65, 1.06)	0.141

a Adjusted for age, sex, drinking and smoking.

A significantly decreased risk of ESCC was observed with pri-miR-124-1 rs531564 GG genotype from Table 4 in patients who were older than 62 years (p = 0.010, OR = 0.52, 95% CI = 0.32–0.86), no drinking (p = 0.004, OR = 0.41, 95% CI = 0.23–0.75) or no smoking (p = 0.026, OR = 0.43, 95% CI = 0.21–0.91). Female patients with miR-124-1 rs531564 GG genotype were associated with a borderline statistically decreased ESCC risk (p = 0.051, OR = 0.36, 95% CI = 0.13–1.01).

As shown in Table 5, significant associations between pri-miR-34b/c rs4938723 CC genotype and ESCC risks were found in the following subgroups, older than 62 years (p = 0.016, OR = 0.79, 95% CI = 0.65–0.96), males (p = 0.033, OR = 0.84, 95% CI = 0.71–0.99) and smokers (p = 0.026, OR = 0.81, 95% CI = 0.68–0.98). Drinkers and non-drinkers both showed a borderline statistically decreased ESCC risk (p = 0.056 and p = 0.062).

### MDR Analyses to the ESCC Risk of Five SNP Sites and Non-genetic Factors

Using the MDR analysis and including these SNPs and non-genetic factors, the results were presented in Table 6. We found that all models have significant test results (p≤0.05), and the three-factor model (smoking, drinking, gender) had a maximum cross-validation consistency (100%), a higher testing balanced accuracy and a minimum p-value in the significant test, which was the optimal model.

**Table 6:** MDR analysis for the ESCC risk in genetic and non-genetic factors.

Model	Testing Accuracy	Cross-Validation Consistency	p
Smoking	0.5864	10/10	0.0070
Smoking Drinking	0.5923	10/10	0.0031
Smoking Drinking Gender	0.5921	10/10	0.0035
mir-27 mir-218 mir-34 Drinking	0.5891	5/10	0.0054

## Discussion

In this study, we evaluated the association between five genetic variations in pri/pre-microRNA and the risk of ESCC in a Chinese population. We found that two SNPs, rs531564 in pri-miR-124-1 and rs4938723 in pri-miR-34b/c, were associated with a significant decreased risk of ESCC.

Recent research showed that the microRNA miR-124 might play an important role in tumorigenesis [16]. Some case-control studies have concentrated on the relationship between the rs531564 in pri-miR-124-1 polymorphism and the cancer risk, such as bladder cancer [23] and esophageal cancer [22] (mainly type is EADC). However, the relationship between pri-miR-124-1 rs531564 polymorphism and ESCC risk has not been reported till now.

In this case-control study, we found that the GG genotype of the pri-miR-124-1 rs531564 polymorphism is associated with significantly decreased ESCC risk in the recessive model. These results suggest that the GG genotype in rs531564 is a protective factor for ESCC in the Chinese population.

The molecular basis for this action remains unclear; however, Qi et al. [20] suggested that the GG genotype changes the formation of a ring-shaped structure compared with the CC genotype on the secondary structure of pri-miR-124-1. They also found that mature miR-124 expression in the GG genotype is higher than that in the CC genotype. Moreover, Makeyev et al. [30] showed that miR-124 directly targets polypyrimidine tract-binding protein 1 (PTBP1) mRNA, which encodes a global repressor of alternative pre-mRNA splicing in non-neuronal cells. Their research also indicated that miR-124 expression leads to a decrease in the endogenous PTBP1 protein level. Another study demonstrated that knockdown of PTBP1 expression by siRNA impairs the growth of ovarian tumor cells and diminishes their malignant potential [31], indicating that overexpression of PTBP1 can be an important component of a multistep process of carcinogenesis.

These previous findings and the results of the present study characterize a potential mechanism of pri-miR-124-1 rs531564 in carcinogenesis as follows: First, the rs531564 GG genotype may promote the expression of miR-124, which is a cancer suppressor. Second, miR-124 may subsequently target PTBP1, which acts as an oncogene, and decrease its expression. These processes eventually lower the risk of ESCC.

We observe that pri-miR-124-1 rs531564 GG polymorphism has more significant effects on decreasing ESCC risks in subgroups of elderly persons, females, no drinking and no smoking people. These results indicate that sex, age, smoking and drinking can impact the effect of this SNP site in ESCC genesis. For example, as smoking and drinking are the main risk factors for ESCC, the effect of rs531564 GG type was no longer significant in smoking or drinking group of decreasing the ESCC risks. We noticed that one case-control study showed that rs531564 variant carriers have increased risks for esophageal cancer in Caucasian population [22]. However, the amount of population and ethnicity in Ye’s study are different from our research. These may be the main causes of the differences.

In this study, we also checked the relationship between pri-miR-34b/c rs4938723 C/T polymorphism and the ESCC risk. To our knowledge, this is the first study to investigate the association between this SNP site and the ESCC risk.

There are two miR-34 loci in human genomes, one encoding miR-34a and the other encoding miR-34b/c. Recently, several studies have shown that miR-34b/c may play an important role in tumorigenesis. Some studies have found that miR-34b/c were down-regulated in oral cancer [32] and colorectal cancer [33].

The rs4938723 C/T polymorphism, located within the CpG island of pri-miR-34b/c, were reported to be associated with risks of many kinds of cancer. For example, this SNP site has been reported to be associated with an increased risk of hepatocellular carcinoma and nasopharyngeal carcinoma. These findings suggest that the C allele of pri-miR-34b/c rs4938723 may be a risk factor for the development of hepatocellular carcinoma [24] and nasopharyngeal carcinoma [34].

However, in the current study, we found that in the recessive model, CC genotype of pri-miR-34b/c rs4938723 was associated with a significant decreased risk of ESCC. Several other studies have also examined the relationship between pri-miR-34b/c rs4938723 and the cancer risk, and obtained results similar to ours. For example, compared with the TT genotype, CC genotype of pri-miR-34b/c rs4938723 was found associated with a significant decreased risk of colorectal cancer. In another kind of tumor, intracranial aneurysm, they also observed similar results.

The possible reason for different results may be that the same variation in microRNA plays different roles in different types of cancers.

We also utilized the MDR analysis in order to explore the relation between the high-order multiple-factor interactions and the risk of ESCC. We found that smoking, drinking and sex were the main risk factors that can lead to ESCC. These results suggest that among the various factors, non-genetic factors, such as smoking and drinking, are the main factors that can lead to cancer risk. This result suggests that lifestyle plays an important role in carcinogenesis. This can also partly explain why there are differences between subgroups that have distinct smoking and drinking status in the stratified analysis.

In summary, our study examined the relationship between five SNPs in microRNA and ESCC risk. We finally found that GG genotype in pri-miR-124-1 and CC genotype in pri-miR-34b/c may decrease risks of ESCC in a Chinese population. These results suggest that some microRNAs may play an important role in ESCC genesis. Our findings enhance the current understanding of the mechanism of ESCC.

## Supporting Information

Table S1 Primers used in SNP genotyping procedure.
(DOC)
