# Supplementary material for: Pri-miR-124 rs531564 and pri-miR-34b/c rs4938723 Polymorphisms Are Associated with Decreased Risk of Esophageal Squamous Cell Carcinoma in Chinese Populations
Source: PLoS One. 2014 Jun 19;9(6):e100055. doi: 10.1371/journal.pone.0100055 (PMC4063769; doi:10.1371/journal.pone.0100055)
Supplement: Table S1 — Primers used in SNP genotyping procedure. (DOC) [file pone.0100055.s001.doc]

**Table S1. Primers used in SNP analysis**

| **miR-26-1 rs7372209** |  |
| --- | --- |
| Primer-forward | GCCCAATGGCATAGCAAGAATTAGGAGA |
| Primer-reverse | CTCTTGGCTCCTGTGGCTTCATTCC |
| Primer-extend | TTTTAGTCATGCTTACAGTCACGTGGTAC |
| **miR-27 rs895819** |  |
| Primer-forward | ACTTAGCCACTGTGAACACGACTTGG |
| Primer-reverse | ATTGCCAGGGATTTCCAACCGACC |
| Primer-extend | (T)_12_CTGCTTGTGAGCAGGGTCCAC |
| **miR-124-1 rs531564** |  |
| Primer-forward | CCCACATACAATGAGTCCTGAGCCC |
| Primer-reverse | CCGTGGGGTGGGGAGGTGTG |
| Primer-extend | (T)_20_AGCTTCTGTTTCTCTCCCTGAGTCT |
| **miR-218 rs11134527** |  |
| Primer-forward | AAGCAGCGTGGAGAAGCGGAAG |
| Primer-reverse | CCGAGTATGGGCATCCAGTGATTTCC |
| Primer-extend | (T)_28_GGAACCCCACTCCTGATACTAATCA |
| **miR-34 rs4938723** |  |
| Primer-forward | CCTCCTCTGGGAACCTTCTTTGACCTA |
| Primer-reverse | ATAGTGAGCCAGGCAGCTTGTTAGTTAC |
| Primer-extend | (T)_34_CCTCTGGGAACCTTCTTTGACCTAT |
